# Analysis of double-emulsion droplets with ESI mass spectrometry for monitoring lipase-catalyzed ester hydrolysis at nanoliter scale

**DOI:** 10.1007/s00216-022-04266-2

**Published:** 2022-08-23

**Authors:** Laura Heiligenthal, Marie van der Loh, Matthias Polack, Maximilian E. Blaha, Susanne Moschütz, Antje Keim, Norbert Sträter, Detlev Belder

**Affiliations:** 1 Institute of Analytical Chemistry, Leipzig University, Linnéstraße 3, 04103 Leipzig, Germany; 2 Institute of Bioanalytical Chemistry, Leipzig University, Deutscher Platz 5, 04103 Leipzig, Germany

**Keywords:** Double-emulsion droplets, Electrospray ionization mass spectrometry, Microfluidics, Lipase-catalyzed reaction

## Abstract

Microfluidic double-emulsion droplets allow the realization and study of biphasic chemical processes such as chemical reactions or extractions on the nanoliter scale. Double emulsions of the rare type (o_1_/w/o_2_) are used here to realize a lipase-catalyzed reaction in the non-polar phase. The surrounding aqueous phase induces the transfer of the hydrophilic product from the core oil phase, allowing on-the-fly MS analysis in single double droplets. A microfluidic two-step emulsification process is developed to generate the (o_1_/w/o_2_) double-emulsion droplets. In this first example of microfluidic double-emulsion MS coupling, we show in proof-of-concept experiments that the chemical composition of the water layer can be read online using ESI–MS. Double-emulsion droplets were further employed as two-phase micro-reactors for the hydrolysis of the lipophilic ester *p-*nitrophenyl palmitate catalyzed by the *Candida antarctica* lipase *B* (CalB). Finally, the formation of the hydrophilic reaction product *p-*nitrophenol within the double-emulsion droplet micro-reactors is verified by subjecting the double-emulsion droplets to online ESI–MS analysis.

**Graphical abstract:**

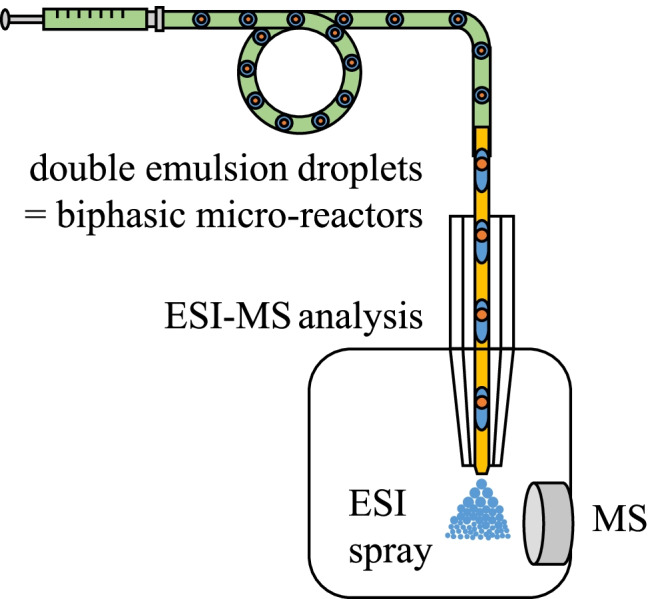

**Supplementary Information:**

The online version contains supplementary material available at 10.1007/s00216-022-04266-2.

## Introduction

Droplet microfluidics offers a bundle of tools that can be utilized to solve current scientific and industrial issues, including the study of (bio-)chemical reactions at the nanoscale [1, 2], single-cell analysis [3–5], high-throughput drug screening [6], and particle synthesis [7]. The technology is based on generating and analyzing highly monodisperse *p*L-*n*L-sized segments embedded in an immiscible carrier phase.

As each droplet represents a discrete compartment and can be individually addressed by manipulating operations including dosing [8, 9], droplet splitting [10], and merging [11], the application of droplets as individual tiny reaction vessels is highly attractive [12]. Reaction parameters such as temperature, reaction time, reagent, and solvent composition can be adjusted and controlled for each droplet individually, enabling the conduction of numerous reactions in parallel and in a high-throughput manner [13–15]. Furthermore, producing these micro-droplets requires only minimal amounts of chemicals and solvents, hence being an economic, environmental friendly, and safe alternative to conventional large-scale reactions [16].

Reaction monitoring inside the droplets can be achieved by analyzing the droplets with either offline or online detection techniques. The online analysis of micro-droplets with electrospray ionization (ESI) mass spectrometry (MS) as a versatile detection technique allows following reaction processes almost in real-time. The monitoring of (bio)chemical reactions performed in micro-droplets with ESI–MS was successfully realized by our group and several others in the past [1, 2, 13, 14, 17–21]. However, the content analysis of micro-droplets by ESI–MS also comes along with limitations that hamper the unrestricted application of this detection technique for general reaction monitoring. Due to the physical basics of the ESI process, polar, volatile solvents, including water, acetonitrile, methanol, and mixtures, and aqueous buffer solutions are suited to achieve a sufficient ionization and later detection of the analytes of interest [21]. ESI–MS analysis of biphasic systems [22–24] has been described. However, ESI–MS analyses of compounds in non-polar, oil-like solvents are challenging. There is unfortunately still no sufficient method to investigate chemical processes in two immiscible phases on a single-droplet level by ESI–MS [25]. In the classical macroscopic laboratory, extraction methods such as separating funnels are often used to convert reaction mixtures from non-polar organic solvents into ESI–MS compatible solutions. In principle, such a macroscopic liquid–liquid extraction can also be done on a nanoliter droplet level using double-emulsion droplets (DEDs).

DEDs are emulsions of emulsions or “droplets in droplets.” A DED is composed of one or multiple inner core droplets of one phase embedded in a larger droplet composed of a second immiscible shell phase. The third phase is a continuous carrier phase that separates the individual DEDs. Two types of DEDs are commonly distinguished: oil-in-water-in-oil (o/w/o) and water-in-oil-in-water (w/o/w) droplets, with the latter being the most investigated. DEDs can be generated by further emulsifying simple water-in-oil (w/o) or oil-in-water (o/w) droplets in a third phase, which is aqueous in the first case and composed of oil in the second case. An important aspect concerns the surface properties of the droplet generators to ensure wetting of the continuous phase and to avoid wall contact of the droplet phase. This aspect is important in the material selection of microfluidic devices. The interfacial areas between the liquid phases are stabilized by common ionic and/or non-ionic surfactants including SDS [26, 27], PEG [28], PVA [29–31], Tween 20 [32]), SPAN 80 [33, 34], and surface-active polymers and/or particles [35, 36]. For the measurement with ESI–MS, a suitable surfactant should be considered that does not negatively interfere with the ionization process. In our work, we focused on the surfactants Triton-X-100 and 1-dodecyl-β-D-maltoside. This approach, which will be explored in this paper, would pave the way to investigate biphasic reactions on a nanoliter scale. Interesting applications are bio-transformations that take place in an organic–aqueous solvent mixture and other processes in double emulsions like material synthesis, phase transfer catalysis, or whole-cell biocatalysis.

This study focuses on biocatalysis, where the hydrophobic substrates are dissolved in an organic solvent, while the aqueous phase contains the enzyme [37]. As reported in the literature, diverse approaches, including biphasic slug- [38], droplet-flow reactors, and bicontinuous microemulsion reactors [38], were employed to conduct biphasic reactions on a miniature scale. Miniaturization of this approach would not only result in lower resource consumption but also in higher surface-to-volume ratios and thus improved reactivity. Furthermore, it could open a way to study catalytic transformations of single cells [5] and single particles also in non-polar solvents with MS.

However, this type of analysis of o_1_/w/o_2_ double-emulsion droplets with ESI–MS is still uncharted scientific territory and will be explored here for a lipase-catalyzed transformation at the nanoliter scale.

## Materials and methods

### Chip-capillary setup

**Fig. 1:**
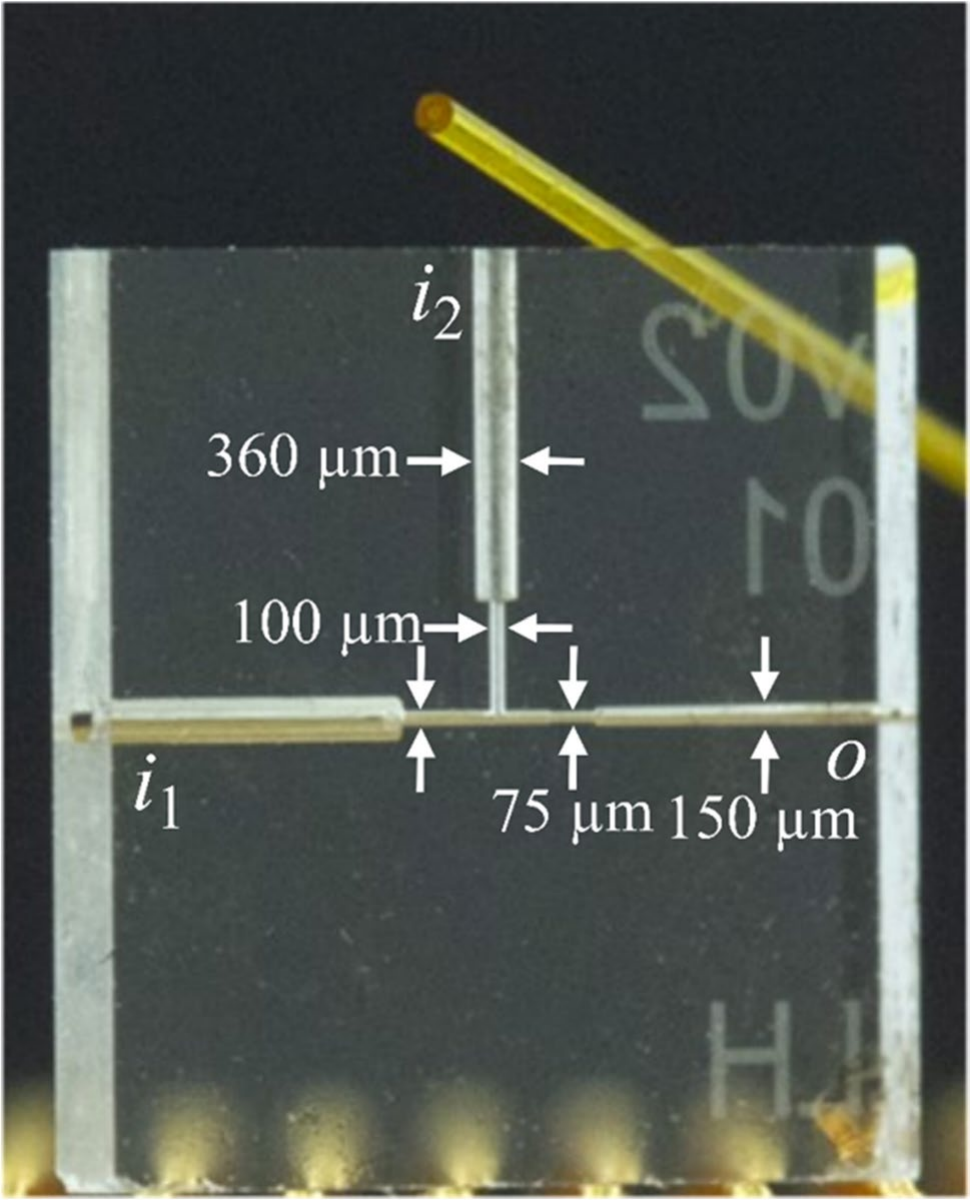
Image of the fused silica glass chip used in the chip-capillary setup. The two inlet channels *i* have a diameter of around 360 µm to fit a 360 µm FS capillary. The outlet channel *o* has a diameter of around 150 µm for smaller FS capillaries. To generate the droplets at the T-junction, a diameter of around 100 µm is implemented. A short channel with a diameter of around 75 µm connects the T-junction with the outlet channel *o.* A microscopic picture of the T-junction is seen in Fig. 2. For comparison purposes, a FS capillary (OD 360 µm ID 100 µm) is shown in the upper part of the image

**Fig. 2:**
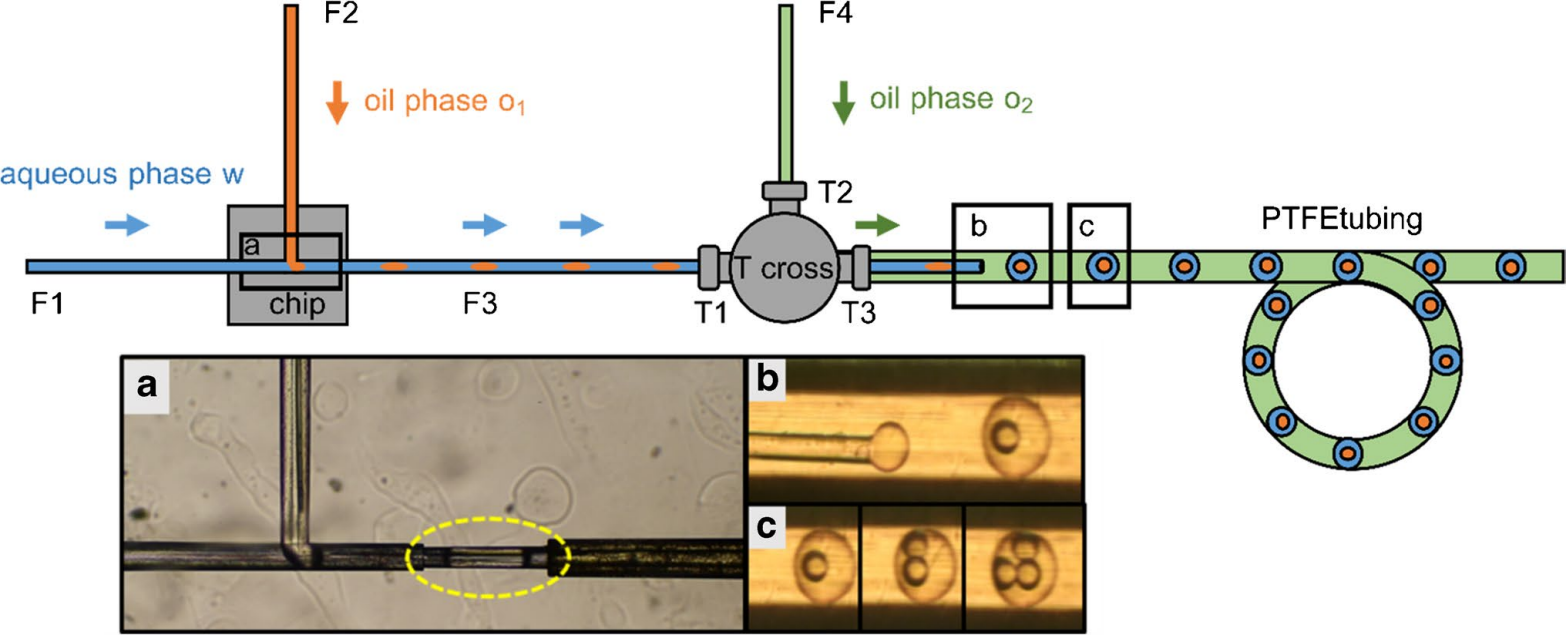
Schematic of the chip-capillary setup utilized for the generation of double-emulsion droplets. **a** First emulsification step: *n-*dodecane segments (highlighted in yellow) stabilized by the surfactant Triton-X-100 are formed at the glass chip’s hydrophilic T-junction in a continuous aqueous phase. **b** The *n*-dodecane segments are transported through a FS capillary to a SST tee-piece at which the second emulsification step takes place. Double-emulsion droplets are generated inside of a PTFE tubing. FC-40 oil with the surfactant PicoSurf is utilized to emulsify the aqueous phase containing the core *n-*dodecane segments. The generated droplets were stored in the PTFE tubing before being coupled to ESI–MS using a CE ESI–MS sprayer interface. **c** By adjusting the pump pressures, double- or multiple-emulsion droplets with an average volume of 14 nL for the shell phase and 1.8 nL for the core phase could be generated

The setup comprised a monolithic glass chip with a T-junction for droplet generation and a stainless steel (SST) tee-piece (1 mm bore size, inlets for 1/16″ fittings). A schematic of the chip-capillary setup is shown in Fig. 2. The monolithic fused silica (FS) glass chip (Siegert Wafer GmbH, Aachen, Germany) utilized in the setup and depicted in Fig. 1 was fabricated in-house by selective laser-induced etching (SLE). The two inlet channels *i* of the chip had diameters of around 360 µm to allow the insertion of 360 µm outer diameter (OD) FS capillaries. The inlet channels were interconnected via a T-junction. The T-junction and the interconnecting microfluidic channels had diameters of around 100 µm. The chip outlet channel *o* had a diameter of around 150 µm to allow the insertion of a 150 µm OD 75 µm inner diameter (ID) FS capillary. A short channel with a diameter of around 75 µm connected the T-junction with the outlet channel *o*.

Three FS capillaries F1, F2, and F3 (F1, F2, OD 360 µm ID 100 µm; F3, OD 150 µm ID 75 µm) (CS-Chromatographie Service, Langerwehe, Germany), were cut to appropriate lengths (15.3 cm, 14.5 cm, and 7.4 cm for F1, F2, and F3). The capillaries’ ends were grinded using lapping paper (9 µm grain size) (3 M, Maplewood, Minnesota, USA). The ends of F1 and F2 were glued in the two chip inlet channels, whereas the end of F3 was glued in the chip outlet channel using epoxy glue EPO-TEC 730 and EPO-TEC 730–110 in a mass ratio 1:1 (EPO-TEC, Billerica, USA). The capillary F3 connected the chip and the tee-piece (Fig. 2). The free end of F3 was inserted in the tee-piece’s left inlet (Fig. 2, T1) and moved through the bore of the tee-piece until approximately 1 cm of the capillary protruded from the tee-piece outlet (Fig. 2, T3). Next, F3 was dead volume-free connected to the tee-piece inlet T1 by a sleeve (OD 1/16″ ID 0.007″, Postnova Analytics, Landsberg am Lech, Germany) and a 1/16″ PEEK fitting. To prevent the aqueous phase from adhering to the outer capillary wall during the droplet formation process, the polyimide coating on the protruding end of F3 was burned off with a lighter. Next, the capillary was coated with a silane by inserting the capillary’s end in a 1 vol% solution of trichloro(*1H,1H,2H,2H*-perfluorooctyl)silane (97%, Sigma-Aldrich, Schnelldorf, Germany) in Novec 7500 oil. Then the end of F3 was inserted into the end of a PTFE tubing (OD 1/16″ ID 300 µm, approximately 1 m in length) and aligned with the center of the tubing. A 1/16″ PEEK fitting connected the PTFE tubing with the tee-piece outlet T3. The double-emulsion droplets were generated inside of the PTFE tubing.

The liquid phases were delivered from sample reservoirs through three FS capillaries L1, L2, and L3 (L1, L2, OD 360 µm ID 100 µm; L3, OD 360 µm ID 250 µm) to the setup using a low-pressure based pump (MFCS-EZ, Fluigent, Jena, Germany). The sample reservoir containing the core oil phase o_1_ was a glass vial (Rotilabo-Kurzgewindefläschchen ND9, 1.5 mL, Carl Roth GmbH + Co KG, Karlsruhe, Germany). The sample reservoirs containing the aqueous phase w and the oil phase o_2_ were plastic vials (MicrewTube with molded ridges, 1.8 mL, self-standing, graduated natural, Simport, Canada). Short pieces of PTFE tubing (OD 1/16″ ID 300 µm) used as sleeves connected the capillaries L1 and L2 with the capillaries F1 and F2. They transported the aqueous phase w and the oil phase o_1_, respectively. The capillary L3 was connected with the tee-piece inlet T2 using a sleeve and a 1/16″ PEEK fitting.

### Generation of double-emulsion droplets

Emulsion droplets of the (o_1_/w/o_2_) type were prepared in two serially connected emulsification steps using the chip-capillary setup (Fig. 2). *n-*Dodecane (VEB petrolchemisches Kombinat Schwedt-BT Erkner, Schwedt, Germany) was used as oil core phase o_1_. The aqueous shell phase w consisted of ultrapure water (TKA Wasseraufbereitungssysteme, Niederelbert, Germany) with 0.1, respectively, 0.2 wt% Triton-X-100 (Carl Roth, Karlsruhe, Germany). The oil phase o_2_ was composed of the fluorocarbon oils Novec 7500 (> 99%, 3 M, Neuss, Germany) or FC-40 (> 99%, Ionic Liquid Technologies Io-li-tec, Heilbronn, Germany) with 0.5, respectively, 1.0 vol% of the surfactant PicoSurf (Sphere Fluidics, Cambridge, UK). The first emulsification step took place at the chip’s T-junction. Oil-in-water (o_1_/w) segments were generated at the chip’s T-junction when suitable pressures were applied to the sample reservoirs. The aqueous phase w transported the o_1_ droplets through the capillary F3 towards the tee-piece. The continuous oil phase o_2_ was delivered through the tee-piece inlet T2. The second emulsification step took place approximately 1 cm behind the tee-piece outlet T3 at the free end of the capillary F3, which was aligned in the PTFE tubing. A co-flow was established between the o_1_/w droplet stream inside the capillary F3 and the continuous oil phase o_2_ in the PTFE tubing. When the o_1_/w droplet stream left the tip of capillary F3, the aqueous phase was emulsified by the continuous oil phase o_2_, resulting in the formation of double-emulsion droplets within the PTFE tubing. Double-emulsion droplets with a single or multiple o_1_ core droplets were generated depending on the applied pressures. In addition to the chip-capillary-based DED generator developed here, a 3D-printed nozzle for double droplet production was also used for comparative purposes, which was supplied by the company (Fluigent, Le Kremlin-Bicêtre, France). A respective photograph of the setup and the generated DEDs is given in the supporting information in Fig. S4.

For the observation of the DEDs, the setup was positioned on top of an inverted microscope (Olympus IX 50). In addition, a Canon EOS 750D camera and a 4 × microscope objective (Olympus, Plan N, 4x/0.10) were utilized for taking photos and recording movies.

### ESI–MS analysis of double-emulsion droplets

The MS experiments were conducted on an Ion Trap XCT mass spectrometer (1100 Series, LC/MSD Trap, Agilent Technologies Inc., City of Santa Clara, CA, USA). The mass spectrometer was controlled by the software LC/MSD Trap Software 5.3 (Bruker Daltonics GmbH, Bremen, Germany). The software Compass DataAnalysis (version 4.2, Bruker Daltonics GmbH) was utilized to obtain the total and extracted ion currents (TICs, respectively, EICs) from the recorded MS data files. Graphs were created using the software OriginPro 8G (OriginLab Corporation, Northampton, MA, USA).

**Fig. 3:**
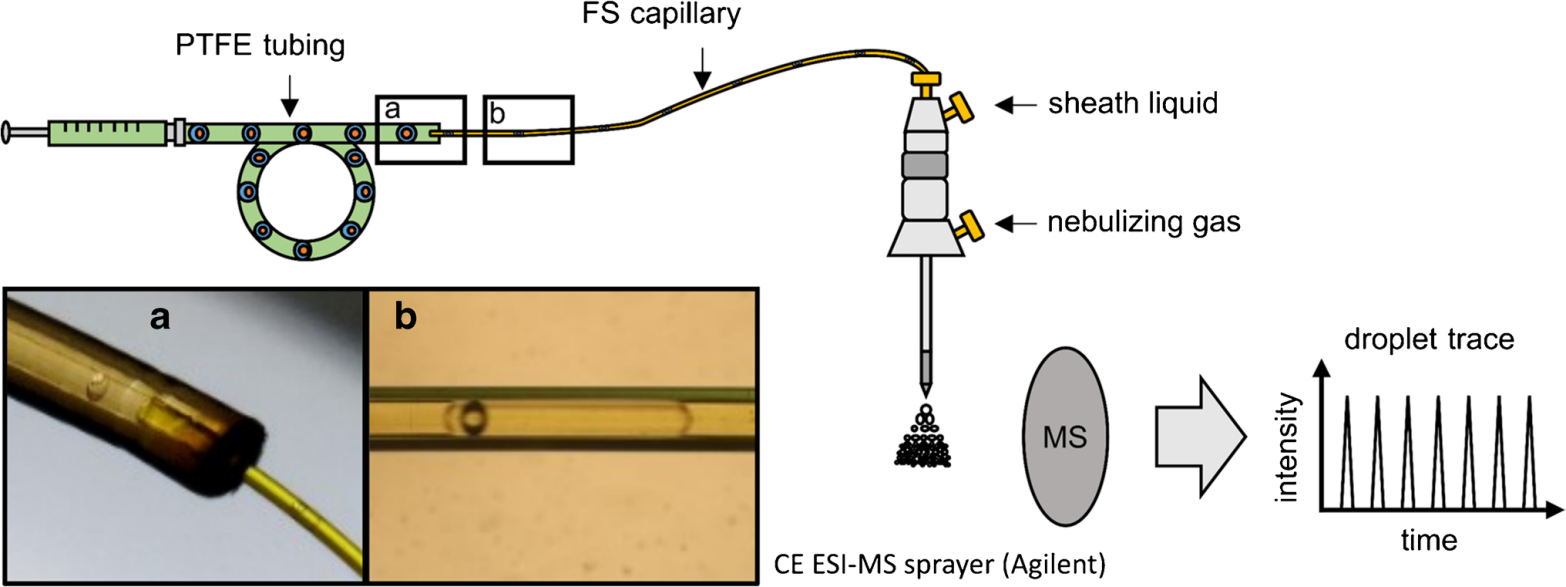
Schematic drawing of the setup with photos of the droplet transfer for the analysis of o_1_/w/o_2_ double-emulsion droplets with ESI–MS. The droplets were transferred from the PTFE tubing (OD 1/16’’ ID 300 µm), in which they were generated and stored, into a silanized FS capillary (OD 360 µm ID 250 µm, respectively 150 µm), which was inserted into the CE ESI–MS sprayer and functioned as emitter. **a** Microscopic picture of a DED transitioning from the PTFE tubing into a FS capillary with ID 250 µm. **b** Out-of-focus microscopic picture of a DED inside a FS capillary with ID 150 µm

Instead of the standard ESI sprayer, we employed a capillary electrophoresis (CE) ESI sprayer (G1607A, Agilent Technologies, Waldbronn, Germany) schematically depicted in Fig. 3. The CE sprayer was inserted into the ESI chamber in an orthogonal position to the MS inlet. This orthogonal alignment minimizes oil contamination of the MS. A silanized FS capillary (360 µm OD 250 µm ID) was threaded through the CE ESI sprayer until ~ 0.5–1 mm protruded. The silanization was afforded by flushing the FS capillary with Novec 7500 oil and subsequently with a 1 vol% solution of trichloro(*1H,1H,2H,2H*-perfluorooctyl)silane in Novec 7500 oil. An additional effect of silanization is that the oil wets the outside of the capillary and thus is diverted away from the MS, further reducing the risk of oil reaching the MS inlet. The FS capillary’s free end was inserted into one end of the droplet containing PTFE tubing. The other end of the PTFE tubing was connected to a 100 µL gas-tight syringe (ILS, Stützerbach, Germany) filled with Novec 7500 oil. The syringe was driven by a low-pressure syringe pump (kD Scientific, Holliston, MA, USA). We applied a flow rate of 1 µL/min to move the droplets from the PTFE tubing through the FS capillary to its tip, at which the electrospray was created. A sheath liquid was employed to electrically contact the aqueous phase of the droplets at the FS capillary’s tip. It consisted of methanol (HPLC gradient grade, VWR Chemicals, Darmstadt, Germany) and ultrapure water in the ratio of 1:1 (vol%/vol%) and was delivered by an HPLC high-pressure pump (Knauer, Berlin, Germany). A sheath liquid flow rate of 50 µL/min was used for the proof-of-concept measurement of the double-emulsion droplets containing adenosine and venlafaxine. The sheath liquid flow rate was set to 0 *µ*L/min for measuring the double-emulsion droplets containing the substrate *p-*NPP and lipase to avoid diluting the droplets.

## Results and discussion

### Development of a chip-capillary setup to generate double-emulsion droplets

We developed a straightforward chip-capillary setup, which allowed us to generate regularly spaced DEDs of the o_1_/w/o_2_ type in a two-step emulsification process and to couple them for the first time with ESI–MS. The generation of DEDs in a two-step emulsification process in microfluidic chips or capillary devices is a standard procedure [39–41]. However, many works focus on the fabrication of w_1_/o/w_2_ DEDs with thin or even ultrathin shells due to increased stabilities of the droplets, and relatively high surfactant concentrations (often between 1–10 wt%) are utilized [28, 33, 42–46]. While high detergent concentrations facilitate the production of double droplets, they must be avoided for droplet-ESI–MS coupling as they cause signal suppression and contamination of the MS [47]. In initial attempts, preparing stable o_1_/w/o_2_ DEDs with relatively large aqueous shells using low surfactant concentrations proved challenging. Our setup comprised a microfluidic glass chip connected by an FS capillary to an SST tee-piece. The DEDs were generated in a PTFE tubing connected to the tee-piece. In preliminary tests with *n-*dodecane used as oil phase and the detergents Triton-X-100, 1-dodecyl-β-D-maltoside (DDM), 1-octyl-β-D-glucoside (1-OG), and PEG 1000, it was investigated if o_1_/w droplet generation worked reliably (SI Table S1). The experiments showed that the formation of o/w droplets was successful when the aqueous phase was a solution of DDM or Triton-X-100 in purified water with concentrations of at least 0.005 wt% and 0.05 wt%, respectively. However, no o_1_/w droplets were obtained in the presence of 1-OG and PEG 1000 (concentrations of 2 wt% each).

For the formation of the DEDs, the following combination of oil phases and detergents was suitable. *n-*Dodecane was employed as core oil phase o_1_. The shell aqueous phase w consisted of water with the surfactant Triton-X-100, and the oil phase o_2_ was composed of the fluorocarbon oils FC-40, respectively, Novec 7500 in combination with the surfactant PicoSurf.

The﻿﻿ first emulsification﻿ step took place at the T-junction of the hydrophilic fused silica glass chip. Following the procedure described in the section *Materials and methods*, oil-in-water (o_1_/w) segments with a plug shape were generated at the T-junction in squeezing mode (Fig. 2a). The o_1_/w plugs were transported through the hydrophilic capillary F3 towards its tip, which was aligned in the PTFE tubing. Regularly spaced o_1_/w/o_2_ droplets were obtained within a second emulsification step, which took place at the tip of the capillary F3 inside of the hydrophobic PTFE tubing (Fig. 2b). The second emulsification step will be described in more detail subsequently. A second oil phase o_2_ was introduced via the inlet T2 of the SST tee-piece to establish co-flow between the o_1_/w plugs in capillary F3 and the oil phase o_2_. A droplet of the aqueous phase was formed at the tip’s end of F3, into which one or multiple incoming o_1_ droplets were inserted. The aqueous shell phase was emulsified by the oil phase o_2_ when the shear forces acting on the aqueous phase exceeded a particular value. Then the aqueous phase detached from the capillary’s tip, and an o_1_/w/o_2_ double-emulsion droplet was formed.

We could generate double-emulsion droplets with varying core *n-*dodecane oil droplets (Fig. 2c). The number of core oil droplets could be tuned by changing the pressures applied to the phases o_1_ and w while keeping the pressure applied to the continuous oil phase o_2_ constant. With increasing pressure ratio p(o_1_)/p(w), an increasing number of core oil droplets encapsulated in a single DED was observed. In contrast, a reduction of p(o_1_)/p(w) resulted in DEDs with fewer core o_1_ droplets. By adjusting the pressure applied to the continuous phase o_2_, the overall size of the DEDs and the oil spacing between them could be modified. The encapsulation efficiency and thus the number of core droplets slightly varied between the experiments due to small-pressure instabilities of the setup. The DEDs had an average total volume of 14 nL. The volume of the *n-*dodecane core droplets was on average 1.8 nL. As video S1 shows, 30 two-core DEDs, 1 one-core DED, and 2 three-core DEDs were generated within 1 min. Therefore, we can state that our setup is very flexible concerning the generation of DEDs with different droplet sizes and numbers of core droplets. To improve the robustness, hydrophobic materials like PMMA, COC, or PDMS could be implemented instead of the metal T-cross to build the second T-junction. We have also tested a DED generator based on a 3D-printed nozzle that has recently become available on the market. They promise better reproducibility and robustness due to the well-defined nozzle, arrangement. These comparative data, shown in Fig. S4, demonstrate that this commercial DED generator can indeed improve the reproducibility of droplet generation and thereby the robustness of the system. However, it had the disadvantage of limiting fluidic flexibility in the development of the method due to the fixed nozzle dimensions. Therefore, we mainly used the chip-capillary approach for further studies.

An important aspect of method development was the optimization of the surfactant concentration. To stabilize the *n*-dodecane droplets, at least 0.05 wt% Triton-X-100_._ was needed. Lower surfactant concentrations were not sufficient for the formation of *n-*dodecane droplets. We utilized 0.1 and 0.2 wt% Triton-X-100 for our experiments. The generated o_1_/w/o_2_ droplets were stored in a PTFE tubing before being analyzed with ESI–MS. For higher production levels and subsequent MS screening at higher throughput, a longer tubing could be utilized in further studies. For very fast reactions, the intermediate storage of DEDs can be omitted, which would allow the integration of the droplet microfluidics and electrospray processes on a single device [18].

### Analysis of the double-emulsion droplets with ESI–MS

We used a commercial CE ESI–MS sprayer as an interface to analyze the generated DEDs with ESI–MS. The CE ESI–MS sprayer was mounted in place of the usual ESI sprayer in an orthogonal position to the MS inlet. We connected the 1/16″ OD 300 µm ID PTFE tubing, in which the DEDs were generated and stored, to a syringe filled with the oils Novec 7500, respectively, FC-40. By applying a flow rate of 1 *µ*L/min to the syringe, the DEDs were transported from the PTFE tubing into a 360 µm OD capillary. The 360 µm OD capillary was inserted into the sprayer and functioned as an emitter.

We tested in the first experiments whether a PTFE capillary (OD 360 µm ID 150 µm) could be used as an emitter. PTFE was chosen as capillary material due to its superior hydrophobic surface properties. We assumed that the hydrophobic inner capillary surface would ensure a smooth transition of the DEDs from the PTFE tubing into the capillary as the aqueous phase would presumably not adsorb to the capillary walls. Indeed, no adsorption of the aqueous shell phase to the capillary walls was observed. However, the occasional splitting of some DEDs in two or more separate droplets was observed. The splitting occurred either when the droplets transited from one PTFE capillary to another or after the DEDs had already traveled some centimeters in the capillary due to shear forces (SI Fig. S2). It is well-known in the literature that DEDs are sensitive to shear forces and droplet splitting, as well as loss of internal droplets [48, 49]. In detail, Chen et al. investigated the hydrodynamics of double-emulsion droplets in shear flow [50]. The authors described four different types of droplet breakup via three mechanisms (necking, end pinching, and capillary instability). Therefore, we used silanized FS capillaries (OD 360 µm ID 250 µm and 150 µm) as emitters in further experiments. We observed that the shape of the DEDs’ aqueous phase was distorted from a sphere to a plug shape during their transition from the PTFE tubing into the capillary (Fig. 3a and Video S2). Despite relatively large distortion, especially when the FS capillary with an ID of 150 µm was used (Fig. 3b), neither droplet splitting nor the loss of core oil droplets was observed.

**Fig. 4:**
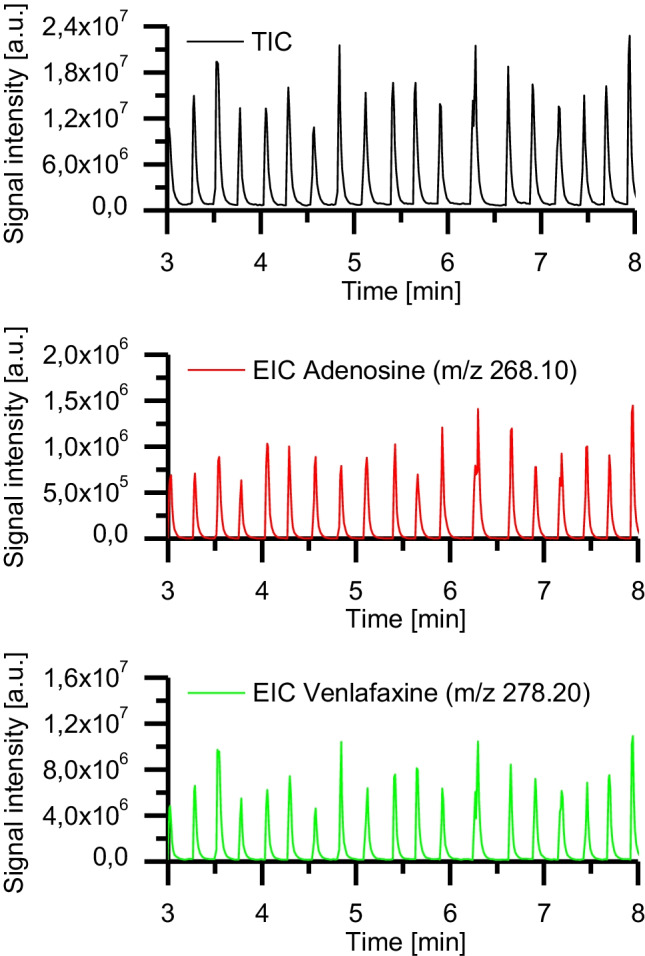
o_1_/w/o_2_ double-emulsion droplets were detected with ESI–MS. Core oil phase o1, n-dodecane; aqueous shell phase w, adenosine and venlafaxine (each 100 µM) in purified H_2_O with 0.2 wt% Triton-X-100; continuous oil phase o_2_, Novec 7500 with 0.5 vol% PicoSurf. Sheath liquid, MeOH/H_2_O (1:1) with a flow rate of 50 µL/min

We investigated in the next step whether detecting model analytes incorporated in the aqueous shell phase of the DEDs was possible with ESI–MS. We generated a series of DEDs that contained mostly two-core n-dodecane o_1_ droplets. The evaluation of 50 droplets revealed that 82% of the droplets contained two, 12% one, and 6% contained no core droplet. The aqueous phase w of the DEDs contained the model analytes venlafaxine and adenosine (100 µM each) in purified H_2_O with 0.2 wt% Triton-X-100. With a shell volume of 14 nL, the amount of venlafaxine and adenosine was 1.4 mmol. The o_1_/w droplets were spaced by a continuous oil phase o_2_ consisting of Novec 7500 with 0.5 vol% PicoSurf. The measurement was performed in ESI-positive mode. Figure 4 depicts the total ion current (TIC) and the extracted ion currents (EICs) of the analytes adenosine (*m/z* [M + H]^+^ = 268.10) and venlafaxine-HCl (*m/z* [M + H]^+^  = 278.20). The TIC and both EICs show a typical droplet pattern which can be obtained as well when injecting droplets made from a single phase [1, 51]. Both analytes could be detected with sufficient signal intensities, although the aqueous phase contained the non-ionic detergent Triton-X-100, which can cause ionization suppression of the analytes [52]. This experiment demonstrates the successful detection of model analytes dissolved in an aqueous phase w of o_1_/w/o_2_ droplets with ESI–MS.

**Fig. 5:**
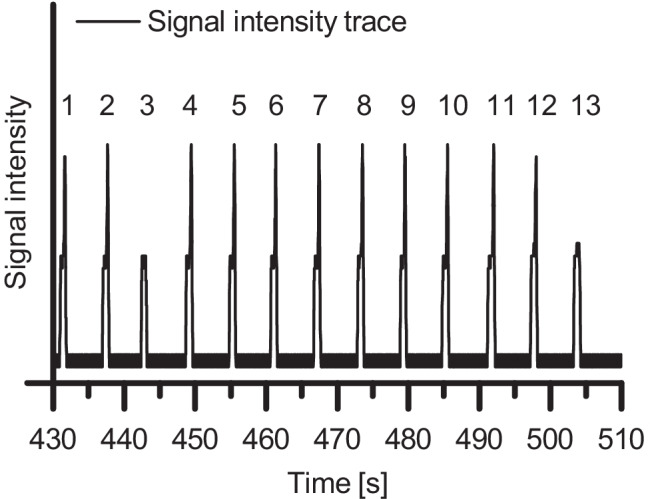
Signal intensity trace of emulsion droplets. Each peak belongs to an emulsion droplet. Each spike on top of a peak originates from a single encapsulated n-dodecane core droplet. The droplets numbered 3 and 13 contain no encapsulated n-dodecane droplet

In subsequent experiments, we investigated how we potentially could identify o_1_ core droplets embedded in the aqueous shell droplet. For the experiments, we utilized a UV/VIS spectrometer (Spectra 100, Spectra-Physics, Stahnsdorf, Germany) equipped with a flow cell, which allowed the insertion of a 360 µm OD FS capillary containing the DEDs. The absorbance values measured by the spectrometer at 405 nm wavelength were converted into voltage values. The voltage values were recorded by a data acquisition device (DAQ USB 6000, National Instruments, Austin, TX, USA) and visualized as a function of time using a self-written LabVIEW program. When the spherical DEDs transited into an FS capillary with OD 360 µm ID 150 µm, the aqueous phase of the DEDs was squeezed. As a result, plugs surrounded by a very thin film of the continuous phase o_2_ were formed (Fig. 3b). As the oil phase o_2_ has a refractive index different from the aqueous phase, this thin film influences the absorbance measurements. This indicated that online UV/Vis spectroscopy is a valuable tool to detect the presence of an embedded core droplet in the aqueous phase (see Fig. 5).

DEDs were generated from the phases *n-*dodecane (o_1_), H_2_O + 0.1% wt% Triton-X-100 (w), and FC-40 + 0.5 vol% PicoSurf (o_2_). The DEDs contained mostly a single *n*-dodecane core droplet. However, small-pressure instabilities generated droplets consisting only of the aqueous phase w (without encapsulated n-dodecane core droplet). The DEDs were pumped through a silanized FS capillary (OD 360 µm ID 150 µm) with a flow rate of 1 *µ*L/min, and their intensity signals were recorded. Each of the 13 peaks belongs to an aqueous droplet separated from each other by the continuous oil phase o_2_. Droplets passing the flow cell caused characteristic intensity peaks due to refraction and reflection phenomena at the liquids’ interfaces. Light, which is significantly refracted or reflected at a liquid–liquid interface, does not reach the detector and thus causes an increase in intensity. An additional liquid–liquid interface exists when an n-dodecane droplet is embedded in the aqueous droplet. Consequently, when the *n-*dodecane droplet passes the flow cell, an additional increase in intensity can be observed in the form of a spike on top of the peak.

The characteristic peak shapes indicate that all aqueous droplets, besides those with numbers 3 and 13 (in Fig. 5), contain a single *n-*dodecane core droplet. Droplets 3 and 13 only consist of the aqueous phase w, showing a single peak belonging to the aqueous droplet phase but no additional spike at the peak’s maximum, which is characteristic of an encapsulated *n-*dodecane core droplet. The spikes were located at each peak’s falling edge since the *n-*dodecane droplets were positioned in the plugs’ back ends and passed the flow cell some seconds later than the plugs’ front ends (Fig. 3b).

We showed with this experiment that the presence, as well as the absence of an n-dodecane core droplet encapsulated in an aqueous shell phase, can be determined online by UV/VIS spectroscopy. This method could also be beneficial in combination with a droplet sorting module, as it would potentially allow to separate droplets differing in the number of encapsulated core droplets from each other. Integrating the UV/Vis spectroscopy would also support the usage of DEDs in a high-throughput manner.

### Lipase-catalyzed ester hydrolysis inside double-emulsion droplets

In our next set of experiments, we aimed to utilize the generated DEDs as tiny reaction vessels for the conduction of a lipase-catalyzed ester hydrolysis reaction taking place at the o_1_/w interface of the individual DEDs. In addition, we wanted to investigate whether the hydrolysis of *p*-NPP would occur in the DEDs and whether the formation of the water-soluble hydrolysis product *p*-nitrophenol could be detected with ESI–MS.

Lipases (EC 3.1.1.3) belong to the group of α/β-hydrolases and the serine hydrolases family [53]. They naturally hydrolyze triglycerides to glycerine and the free fatty acids at an oil/water interface. We chose *Candida antarctica* lipase B (CalB) for our study due to its stability and various use in biocatalytic transformations. CalB shows conformational stability and thus enzymatic activity even in non-polar solvents as for example cyclohexane [54]), toluene ([55], isooctane [56], methyl-tert-butyl ether (MTBE) [57], and dimethyl formamide (DMF) [58]. In contrast to other lipases, CalB does not show a phenomenon known as interfacial activation due to the absence of a lid, which covers the active site entrance in other lipases [59]. Hence, the enzyme does not change its conformation at an interface. CalB is interesting for industrial applications since it can catalyze the kinetic resolution of racemic mixtures yielding optically pure compounds like alcohols [60] and amines [61], as well as asymmetric synthesis.

**Scheme 1:**
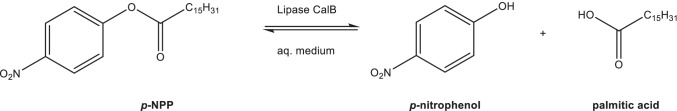
Hydrolysis of *p*-nitrophenyl palmitate (*p*-NPP) yielding *p*-nitrophenol and palmitic acid

We selected the CalB-catalyzed hydrolysis of the ester *p*-nitrophenyl palmitate yielding the UV/VIS active *p*-nitrophenol and palmitic acid as a model reaction (Scheme 1). This reaction is commonly utilized to photometrically determine lipase activity by following the release of *p*-nitrophenol at 405 nm [60]. For the generation of the DED reaction vessels, a 5 mM solution of *p*-NPP in *n*-dodecane and a 0.2% wt% solution of Triton-X-100 in purified H_2_O with CalB (c = 0.068 mg/mL) were prepared. The 5 mM solution of *p*-NPP in *n-*dodecane was used as core oil phase o_1_, and the aqueous solution containing CalB was used as aqueous phase w. The continuous oil phase o_2_ consisted of FC-40 oil with 1 vol% PicoSurf. DEDs were generated with the previously described setup and stored for approximately 2 h 20 min in the PTFE tubing before they were subjected to ESI–MS analysis. As a control, DEDs containing only the substrate dissolved in the core *n*-dodecane droplets but no lipase in the aqueous phase w were generated. The measurements were performed in the negative ionization mode. We found in previous experiments that *p*-nitrophenol can be dissolved well in the aqueous phase and forms in the negative mode the quasi-molecular ion [M-H]^−^ at m/z 138.02. However, the sodium salt of palmitic acid was not water-soluble. Thus, we set the scan range to m/z 100–150, intending to monitor only the hydrolysis product *p*-nitrophenol at m/z 138.02.

**Fig. 6:**
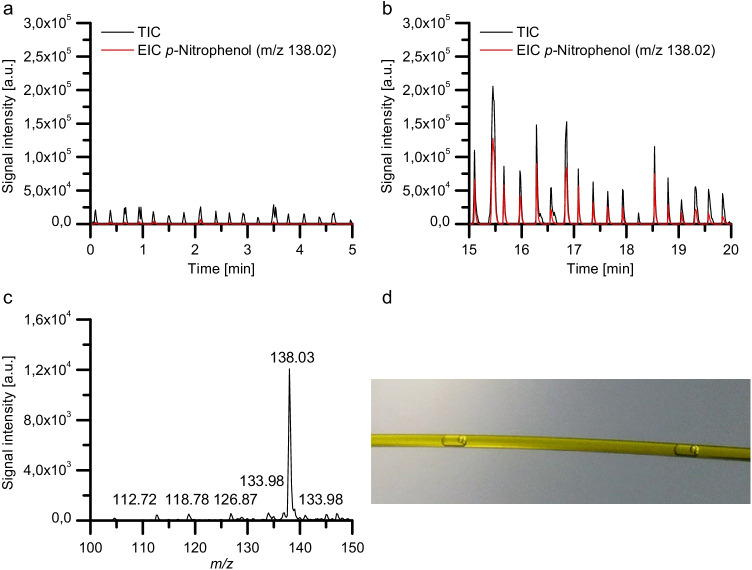
**a**, **b** Mass traces of double-emulsion droplet micro-reactors. **a** shows the mass trace of DEDs containing the substrate p-nitrophenyl palmitate (*p*-NPP, *c* = 5 mM) in the core oil phase o1 and no enzyme in the aqueous phase w. **b** shows the mass trace of DEDs containing the substrate *p*-nitrophenyl palmitate (*p*-NPP, *c* = 5 mM) in the core oil phase o_1_ and the lipase CalB (*c* = 0.068 mg/mL) in the aqueous phase w after an incubation period of approximately 2 h 20 min. **c** depicts the mass spectrum of five DEDs containing 5 mM *p*-NPP and CalB. **d** Microscopic image of DEDs flowing through a FS capillary. Composition of phases: o_1_, *n*-dodecane; w, purified H_2_O with 0.2 wt% Triton-X-100 ± CalB; o_2_, FC-40 with 1 vol% PicoSurf. The droplets were sprayed with a flow rate of 1 *µ*L/min from a silanized FS capillary (OD 360 µm ID 250 µm) inserted into the CE ESI–MS sprayer

Figure 6 shows the TIC and the EIC at m/z 138.02 for DEDs containing substrate but no CalB (a, control) respectively for DEDs containing substrate and CalB (b) after they were incubated in the PTFE tubing for around 2 h 20 min. Figure 6c depicts the mass spectrum recorded of DEDs containing substrate and CalB. The mass spectrum was obtained by averaging the TIC of five consecutive DEDs.

As depicted in Fig. 6a, *p*-nitrophenol was not detected with significant signal intensities in the absence of CalB. However, significant increases in the TIC and EIC at m/z 138.02 were recorded for DEDs containing CalB after an incubation period of around 2 h 20 min. With an intensity of about 1.2 × 10^4^ counts, the peak at m/z 138.02 dominates the mass spectrum, demonstrating the formation of the hydrolysis product *p*-nitrophenol within the individual DEDs. This is comparable to a 400 µL reaction volume and direct injection of the water phase in ESI–MS (seen Fig. S6).

## Conclusion

We presented in this work a straightforward chip-capillary setup, which enabled the generation of uniform, regularly spaced double-emulsion droplets in a two-step process. This was combined with a capillary sheath-flow interface to realize the first analysis of double-emulsion droplets by ESI–MS. The double-emulsion droplets were prepared and stored in a PTFE tubing to facilitate prolonged incubation and reaction times for a lipase-catalyzed ester hydrolysis reaction. After an incubation period, the formation of the hydrolysis product in the droplets could be verified by ESI–MS.

Here, online UV/VIS spectroscopy proved to be a very valuable approach for monitoring the bypassing DEDs. This double-emulsion droplet MS approach allows for studying biphasic reactions at the nanoliter scale. While here we stored the double-emulsion droplets in a capillary intermediate to monitor a slow lipase-catalyzed ester cleavage reaction, this can be avoided for fast reactions and high-throughput screening applications.

## Supplementary Information

Below is the link to the electronic supplementary material.

Supplementary file1 (PDF 763 KB)

Supplementary file2 (MP4 12143 KB)

Supplementary file3 (MP4 9335 KB)
