# Supplementary material for: Analysis of double-emulsion droplets with ESI mass spectrometry for monitoring lipase-catalyzed ester hydrolysis at nanoliter scale
Source: Anal Bioanal Chem. 2022 Aug 23;414(23):6977–87. doi: 10.1007/s00216-022-04266-2 (PMC9436884; doi:10.1007/s00216-022-04266-2)
Supplement: Supplementary file 1 — Supplementary file1 (PDF 763 KB) [file 216_2022_4266_MOESM1_ESM.pdf]

Analysis of double emulsion droplets with ESI mass spectrometry for monitoring lipase catalyzed ester hydrolysis at nanoliter scale

Laura Heiligenthal[1#], Marie van der Loh[1#], Matthias Polack[1], Maximilian E. Blaha[1], Susanne Moschütz[2], Antje Keim[2], Norbert Sträter[2], Detlev Belder[1*]

[1] Institute of Analytical Chemistry, Leipzig University, Linnéstraße 3, 04103 Leipzig, Germany

* ✉ Detlev Belder: belder@uni-leipzig.de

[#] Laura Heiligenthal and Marie van der Loh contributed equally to this work and should be regarded as co-first authors.

[2] Institute of Bioanalytical Chemistry, Leipzig University, Deutscher Platz 5, 04103 Leipzig, Germany

Supplementary Information

Table of Contents

1) Chip fabrication 1
2) Expression and purification of lipase CalB 1
3) Gene coding for the lipase CalB 2
4) Generation of oil-in-water (o/w) droplets 2
5) Splitting of (o_1/w/o_2) double emulsion droplets 3
6) Distortion of the double emulsion droplets' shape 3
7) Reproducibility of droplet diameter 4
8) Fluigent RayDrop double emulsion droplet generator 4
9) Mass spectrometric parameters 5
10) Calibration and reaction monitoring of *p*-nitrophenol 6
11) Direct injection measurement in a 1.5 mL reaction vessel with n-decane 6
12) References 7

1) Chip fabrication

The monolithic tee-piece chip was manufactured by a selective laser-induced etching technique (FEMTOprint f200 aHead P2, Muzzano, Switzerland) followed by a wet chemical etching step in 8 mol L^{-1} potassium hydroxide at 85 °C. First, the microfluidic chip layout was designed via CAD-Software (Autodesk Inventor 2020, San Rafael, California, United States) and processed into the respective machine code with CAM software (Alphacam 2017, Vero Software GmbH, Neu-Isenburg, Germany). Next, a pulsed IR-Laser (1030 nm, 400 fs, 230 nj) was used to inscribe the microfluidic channels with a 20x objective (LMH-20X-1064, N.A. = 0.40, Thorlabs, Bergkirchen, Germany) into a 100 mm fused silica wafer (thickness = 1000 µm, Siegert Wafer GmbH, Aachen, Germany).

2) Expression and purification of lipase CalB

The lipase CalB was expressed and purified with an N-terminal Asp_5-tag and a C-terminal His_6-tag adopting previously published procedures [1], [2]. The Asp_5 sequence enhances soluble expression and secretion of the enzyme. The gene coding for the amino acid sequence specified in Figure S1 was synthesized by ThermoFisher (GeneArt) with codon optimization for *E. coli* expression and *Nco*I and *Xho*I restriction cleavage sites at the 5' and 3' ends, respectively. The synthesized gene was inserted into the expression vector pET26b using these

restriction sites. The construct is expressed with a pelB signal peptide from the pET26b plasmid for secretion into the cell medium. *E. coli* BL21(DE3) cells were transformed with the expression plasmid. For preparative expression, the cells were grown at 37°C in LB medium to an OD600 of 0.6, and expression was induced with an isopropyl-β-D-thiogalactopyranoside (IPTG) concentration of 0.2 mM. The culture was incubated at 20 °C and 220 rpm overnight. On the following day, the cells were pelleted for 30 min at 5000x g. The supernatant was supplemented with 20 mM NaH_2PO_4 and 15 mM imidazole and applied to a HisTrap FF crude 5 mL column. All chromatography steps were conducted at an Äkta pure (Cytiva) FPLC instrument. After washing with 8 column volumes (CV) binding buffer (20 mM NaH_2PO_4 pH 7.4; 0.5 M NaCl; 15 mM imidazole), the protein was eluted with 5 CV of a linear gradient of binding buffer to elution buffer (20 mM NaH_2PO_4 pH 7.4; 0.5 M NaCl; 500 mM imidazole). Fractions containing CalB were pooled, concentrated to 1.5 mL, and applied to Superdex75 (16/60) size exclusion chromatography using a buffer of 20 mM Tris-HCl pH 8.0 with 200 mM NaCl). Active fractions were pooled and concentrated to a protein concentration of 2.3 $mgmL^{-1}$. Protein concentrations were determined using the absorption at 280 nm (measured at a ThermoFisher NanoDrop 2000c instrument) with a calculated extinction coefficient [3] of 41285 M^{-1} cm^{-1} or 1.175 mL mg^{-1} cm^{-1}.

3) Gene coding for the lipase CalB

```
MADDDDDLPSGSDPAFSQPKSVLDAGLTCQGASPSSVSKPILLVPGTGTTGPQSFDSNWIPLSTQLGYTPCWISP
PPFMLNDTQVNTEYMVNAITALYAGSGNNKLPVLTWSQGGLVAQWGLTFFPSIRSKVDRLMAFAPDYKGTVLAGP
LDALAVSAPSVWQQTTGSALTTALRNAGGLTQIVPTTNLYSATDEIVQPQVSNSPLDSSYLFNGKNVQAQAVCGP
LFVIDHAGSLTSQFSYVVGRSALRSTTGQARSADYGITDCNPLPANDLTPEQKVAAAALLAPAAAAIVAGPKQNC
EPDLMPYARPFAVGKRTCSGIVTPGGSGGSGGHHHHHH*
```

Fig. S1 Sequence of the lipase CalB construct used in this study. The expressed protein possesses an N-terminal Asp_5-tag (red) and a C-terminal His_6-tag (green). Shown in blue is a linker sequence to improve the accessibility of the polyhistidine tag. The gene for expression of this construct was synthesized by ThermoFisher (GeneArt) with codon optimization for *E. coli* expression and *Nco*I and *Xho*I restriction cleavage sites at the 5' and 3' ends, respectively.

4) Generation of oil-in-water (o/w) droplets

Table S1 indicates the detergents and their concentrations for forming stable oil-in-water (o/w) droplets using our chip-capillary set-up.

Tab. S1 Tested detergents and their concentrations sufficient for the formation of stable oil-in-water (o/w) droplets

oil phase o_1	detergent added to the aqueous phase w	detergent concentration	formation of stable o_1/w droplets
n-dodecane	Triton-X-100	≥ 0.05 wt% (800 µM)	yes
		< 0.05 wt%	no
	1-dodecyl-beta-D-maltoside	≥ 0.005 wt% (90 µM)	yes
		< 0.005 wt%	no
	1-octyl-β-D-glucoside	2 wt% (68.5 mM)	no
	PEG 1000	2 wt% (20.0 mM)	no

5) Splitting of (o_1/w/o_2) double emulsion droplets

Figure S2 shows that the double emulsion droplets stayed intact when they transited from the 1/16'' OD 300 μm ID PTFE tubing in a silanized 360 μm OD 150 μm ID FS capillary. Occasionally, however, a DED splits into two or more daughter droplets due to shear forces. This occurred when the DEDs transitioned from the PTFE tube into a non-silanized FS or PTFE capillary or after migrating a few centimeters in these capillaries.

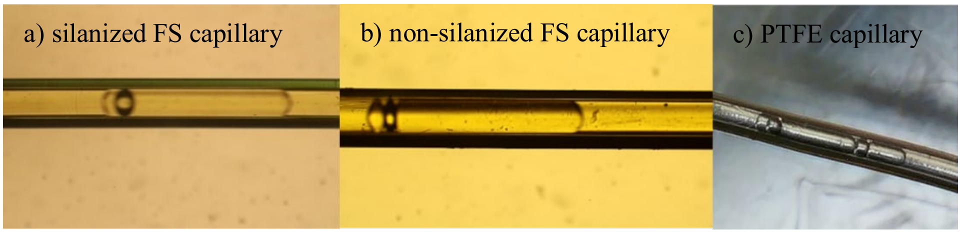

Fig. S2 Intact (a)) and splitted (b, c) double emulsion droplets after their transition from the PTFE tubing (OD 1/16'' ID 300 μm) into FS capillaries respectively a PTFE capillary. All capillaries were 360 μm in OD and 150 μm in ID.

6) Distortion of the double emulsion droplets' shape

Table S2 depicts different combinations of oil and aqueous phases used to generate DEDs and indicates whether the shape of the DEDs was distorted or remained intact.

Tab. S2 Different combinations of oil and aqueous phases were used for the generation of DEDs with a note on the distortion of the droplets' shape.

Oil phase o_1	Aqueous phase w	Oil phase o_2	Distortion of DED shape
n-dodecane	$H_2O_{dest.}$ with 0.2 wt% Triton-X-100	FC-40 oil with 1 vol% PicoSurf	no
n-dodecane	$H_2O_{dest.}$ with 0.2 wt% Triton-X-100 and c (CalB)= 0.068 mg/mL	FC-40 oil with 1 vol% PicoSurf	no
n-dodecane	$H_2O_{dest.}$ with 0.2 wt% Triton-X-100	Novec 7500 oil with 0.5 vol% PicoSurf	no
n-dodecane	$H_2O_{dest.}$ with 0.2 wt% Triton-X-100 and c (CalB)= 0.068 mg/mL	Novec 7500 oil with 0.5 vol% PicoSurf	yes

It becomes clear that the addition of the lipase CalB led to a distortion of the droplets' shape when Novec 7500 oil with 0.5% PicoSurf was used as oil phase o_2 (Figure S3). As Figure S3 shows, the shape of the DEDs was deformed during their downstream transport in the PTFE tubing. While the shape of the aqueous shell phase was approximately spherical when the DEDs detached from the FS capillary, it was elongated as the DEDs traveled through the PTFE tubing, resulting in a plug shape. The shape of the n-dodecane core droplets seemed not to be affected. Furthermore, the regular oil spacing between the DEDs was lost as the Novec 7500 oil flowed alongside the DEDs. Moreover, the encapsulation of the DEDs' core n-dodecane droplets got lost after a certain traveled distance. The n-dodecane droplets seemed to be only attached to the aqueous phase droplet instead of being encapsulated by the aqueous phase. Presumably, the observed deformation in shape was caused by the similar densities of the aqueous shell phase w and the continuous oil phase o_2. Adding lipase to the aqueous shell phase caused an increase in the aqueous phase's density. The deformation in shape was not observed when the oil FC-40

with 1 vol% PicoSurf was used instead of Novec 7500 with 0.5 vol% PicoSurf. FC-40 oil has a higher density (1,9 kg/l [4]) than Novec 7500 oil (1,61 kg/l [5]).

a) b) c) d) e)

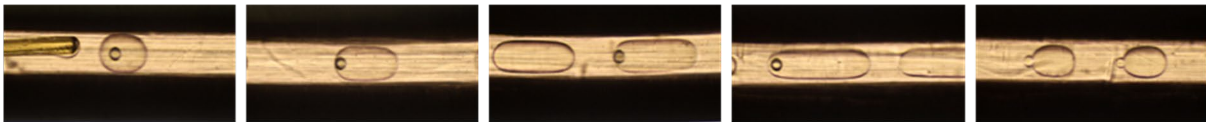

Fig. S3 Deformation in shape of the aqueous shell phase when double emulsion droplets that contain lipase travel through the PTFE tubing. The pictures show different regions in the PTFE tubing. Core oil phase o_1: 5 mM *p*-NPP in *n*-dodecane. Shell phase w: $H_2O_{dest.}$ with 0.2 wt% Triton-X-100 and c (CalB)= 0.068 mg/mL. Outer continuous oil phase o_2: Novec 7500 with 0.5 vol% PicoSurf

7) Reproducibility of droplet diameter

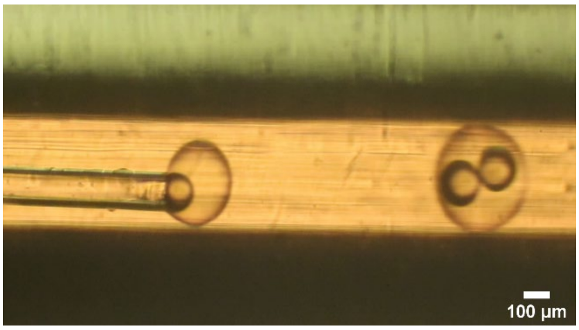

Video S1 Double emulsion generation over 1 min. We got uniform droplets with respectively 1 one-core droplet, 30 two-core droplets, and 2 three-core droplets. Scale bar measured on the 150 µm OD fused silica capillary.

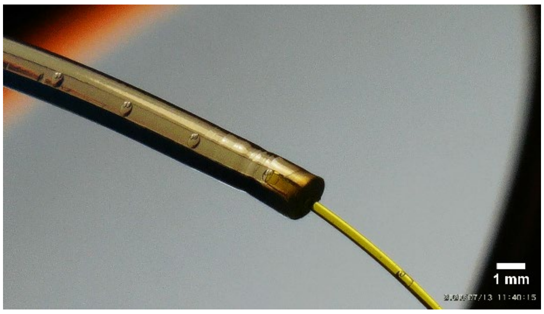

Video S2 Transition of DEDs from the PTFE tubing (OD 1/16'' ID 300 µm) in an FS capillary (OD 360 µm ID 250 µm) towards the MS. Scale bar measured on the 360 µm OD fused silica capillary.

8) Fluigent RayDrop double emulsion droplet generator

In the following, the advantages and disadvantages of the Fluigent RayDrop and our set-up are compared. With our set-up, which consists of the fusion of a T-junction and a co-flow, DEDs can be produced reproducibly with one or multiple core droplets. The advantage of this system is that the sizes of the capillaries and thus also the shell and core droplets can be easily exchanged. The droplets can also be changed due to the different hydrophilic and hydrophobic surfaces. Therefore, even small changes to the system can result in a difference in the droplets. In method development, for example, greater flexibility is desirable.

On the other hand, the commercial Fluigent RayDrop device has a fixed diameter for generating the DEDs. This means there is only a limited volume where the DEDs can be produced reproducibly. The core droplets are often large in volume relative to the shell phase. The generation of smaller droplets (see Fig. S4C) is complex. In addition, droplets with one core are possible with the RayDrop device due to the structure of two simultaneously generating co-flows droplet generators (The nozzle in Fig. S4A and B). On the other hand, the RayDrop system is more robust concerning relocation of the device or exchange of the capillaries.

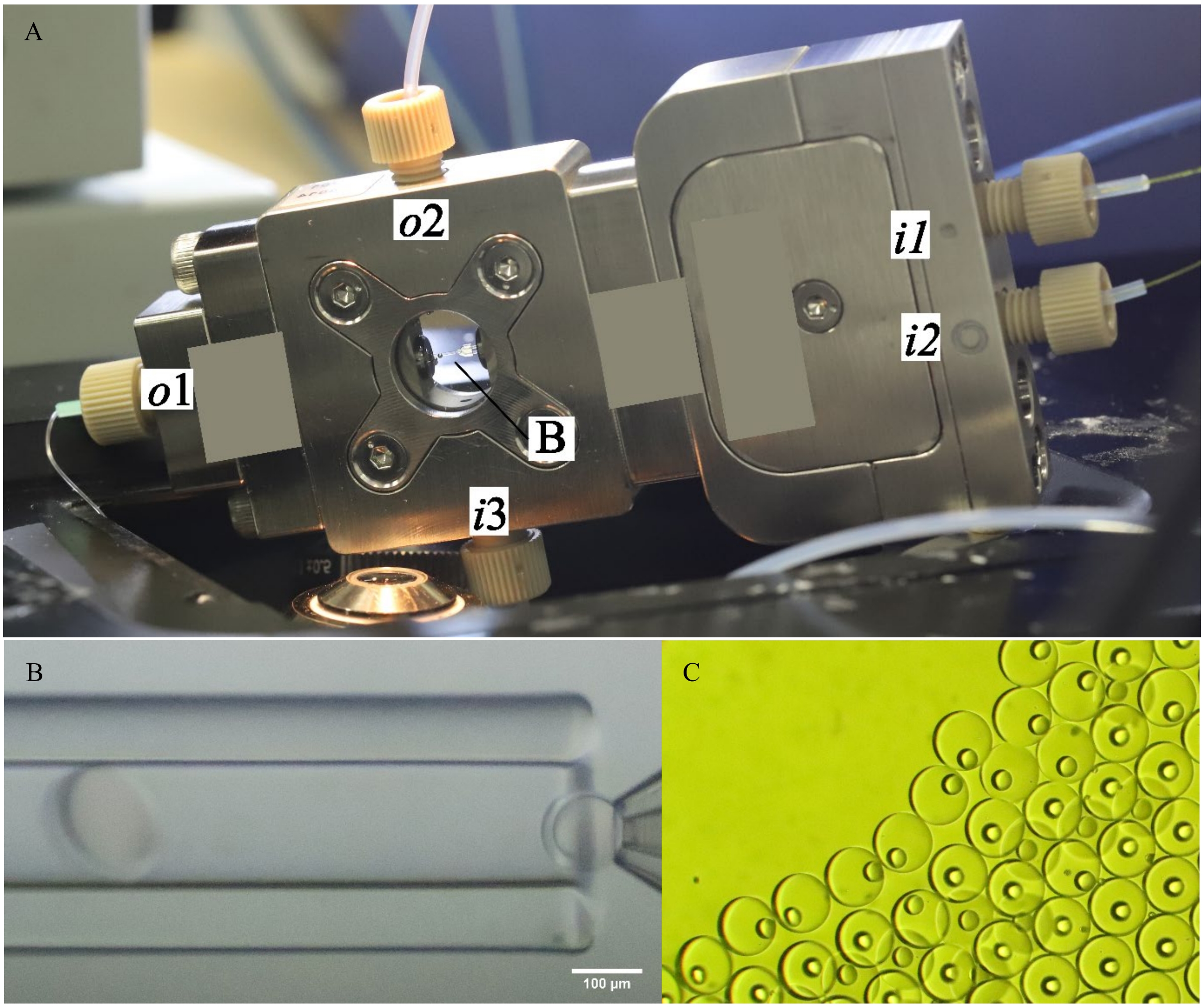

Fig. S4 DEDs produced with the Fluigent RayDrop A: Picture of the RayDrop filled with i-propanol. The inlets *i*1 (core phase), *i*2 (shell phase), and *i*3 (cont. phase) as well as the outlets *o*1 (droplet outlet) and *o*2 (for cleaning) are highlighted. The nozzle with the FS capillary in the chamber can also be seen as a microscopic picture in B: Microscopic picture of a DED generation. The tip of the nozzle can be seen on the right. Phases were Novec 7500 with 1 % 008-FluoroSurfactant (RAN Biotechnologies, Beverly, MA 01915) as cont. phase , shell phase was water with 0.2 mM 1-dodecyl-β-D-maltoside (Cayman Chemical Company, Ann Arbor, Michigan) and 0.02 mM 6-carboxyfluorescein (Sigma-Aldrich Chemie GmbH, Taufkirchen, Germany) and core phase was n-decane (Carl Roth, Karlsruhe, Germany) with 0.2 mM Sudan III (Sigma-Aldrich). Scale bar measured on the 360 µm OD fused silica capillary. C: DED storage and imaging.

9) Mass spectrometric parameters

Measure-ment	Mode of measurement	V (MS capillary) [kV]	Nebulizing gas N_2 [psi]	Dry gas N_2 [psi]	Dry temperature [°C]	Maximum accumulation time [ms]	Scan range [m/z]
M1	positive	-5.5	10	8	250	200	250-350
M2	negative	+5.5	10	8	250	30	100-150

Tab. S3 Mass spectrometric parameters utilized for the measurements M1 and M2

10) Calibration and reaction monitoring of *p*-nitrophenol

For the calibration of *p*-nitrophenol, an internal standard of nitrocatechol was used. Nitrocatechol can mainly compensate the influence of the ESI spray. As seen in Fig. S5A, the signals have a high standard deviation. As known, a high surfactant concentration influences the signals [6]. Therefore, a higher product concentration should be preferable for calibrating our signals. As proof of concept, a reaction monitoring over 120 min showed increased product/internal standard intensity (Fig. S5B). However, after 120 min, the product concentration was still too low for quantitative detection.

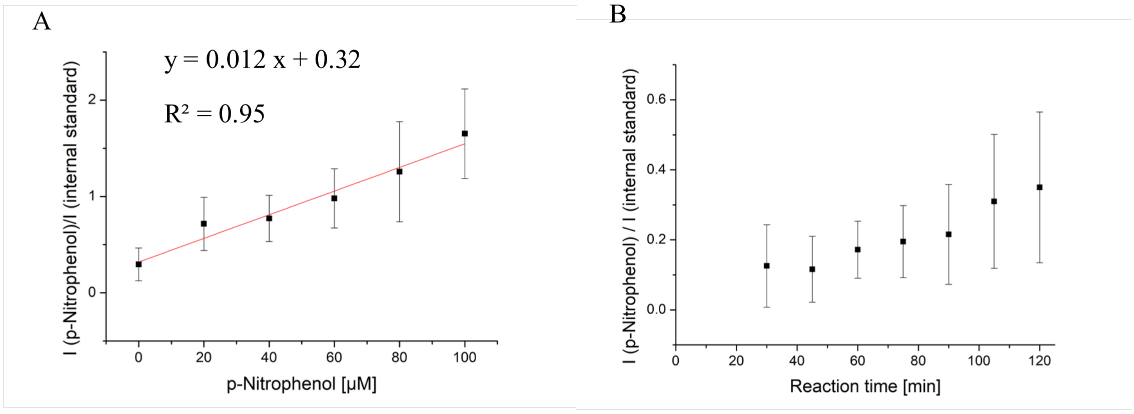

Fig. S5 A: Calibration curve of *p*-nitrophenol and internal standard 10 µM nitrocatechol. More than 60 droplets were analyzed per data point. In B: Reaction monitoring over 120 min of the heterogenic catalyzed reaction by the lipase CalB with the product *p*-nitrophenol. More than 17 droplets were analyzed per data point. The peak height was used for the calculation of both graphs. Furthermore, we used the Grubbs's test to identify droplets that were sprayed irregularly.

11) Direct injection measurement in a 1.5 mL reaction vessel with n-decane

To compare our double emulsion droplet reaction with a direct injection method, we performed the lipase-catalyzed reaction in a 1.5 mL reaction vessel (RotiLab® Microtube). For this purpose, 0.068 mg/mL lipase and 10 µM nitrocatechol as internal standard were diluted in a 10 mM ammonium acetate water solution. Only 10 µM nitrocatechol in water was diluted for the blank solution. The educt 5 mM *p*-nitrophenyl palmitate was dissolved in n-decane and 0.2 mL of it was mixed with 0.2 mL of the water phase. After overnight incubation in a shaker at 37 °C, the product and internal standard in the water phase were detected.

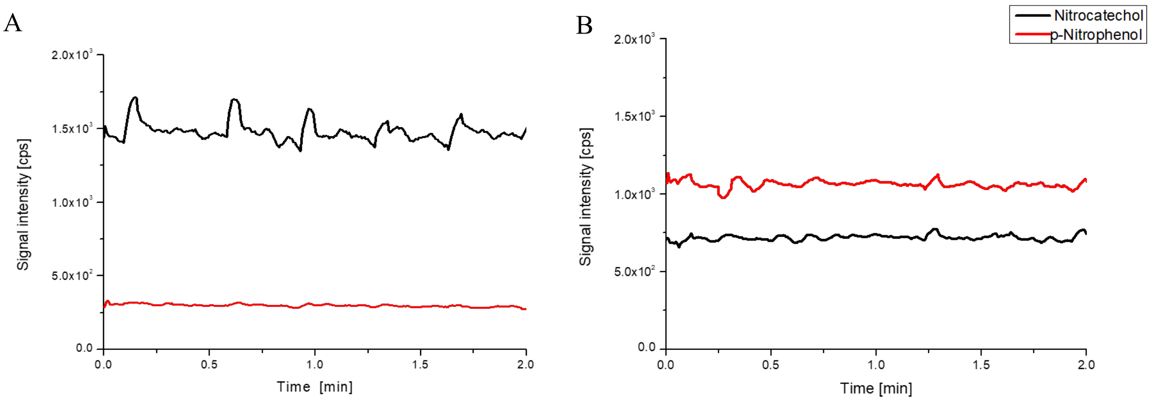

Fig. S6 Direct injection of the water phase form a biphasic solution in a 1.5 mL reaction vessel. A: blank measurement without lipase. 5 mM *p*-nitrophenyl palmitate in n-decane and the internal standard of 10 µM nitrocatechol in 10 mM ammonium acetate. B: An addition of 0.068 mg/mL lipase was added to the water phase. Both solutions were incubated at 37 °C overnight.

The water phase was transferred to a syringe and injected directly with an ESI sprayer (Agilent G1948B ESI Chamber). An MRM mode of an Agilent TQ (Agilent G6495C) was used for the MS measurement. In the MRM mode, the deprotonated *p*-nitrophenol precursor-anion (m/z of 138) is fragmented in the $C_6H_4O_2^{\cdot-}$ product-ion with an m/z of 108 [7]. As shown in Fig. S2, the catalytic lipase reaction generated the *p*-nitrophenol. *p*-Nitrophenol is higher in Fig. S6 B than in the blank solution in Fig. S6 A.

12) References

[1] S.-K. Kim, Y.-C. Park, H. H. Lee, S. T. Jeon, W.-K. Min, and J.-H. Seo, 'Simple amino acid tags improve both expression and secretion of *Candida antarctica* lipase B in recombinant *Escherichia coli*: Simple Tags for CalB Secretion In *E. coli*', *Biotechnol. Bioeng.*, vol. 112, no. 2, pp. 346–355, Feb. 2015, doi: 10.1002/bit.25361.

[2] H.-J. Jung *et al.*, 'Polycationic amino acid tags enhance soluble expression of Candida antarctica lipase B in recombinant Escherichia coli', *Bioprocess Biosyst Eng*, vol. 34, no. 7, pp. 833–839, Sep. 2011, doi: 10.1007/s00449-011-0533-z.

[3] E. Gasteiger *et al.*, 'Protein Identification and Analysis Tools on the ExPASy Server', in *The Proteomics Protocols Handbook*, J. M. Walker, Ed. Totowa, NJ: Humana Press, 2005, pp. 571–607. doi: 10.1385/1-59259-890-0:571.

[4] 3M Deutschland GmbH, '3M Fluorinert Elektronic Liquid FC-40 Sicherheitsdatenblatt'. May 08, 2014. Accessed: Mar. 10, 2022. [Online]. Available: https://iolitec.de/sites/iolitec.de/files/sds/FL-0005-HP%2C%20SDS%20FC-40%20SDB.pdf

[5] 3M Deutschland GmbH, 'Datenblatt 3M Novec 7500 High-Tech Flüssigkeit'. Apr. 09, 2014. Accessed: Mar. 10, 2022. [Online]. Available: https://multimedia.3m.com/mws/media/604414O/3mtm-novectm-7500-engineered-fluid.pdf

[6] Y. Ishihama, H. Katayama, and N. Asakawa, 'Surfactants Usable for Electrospray Ionization Mass Spectrometry', *Analytical Biochemistry*, vol. 287, no. 1, Art. no. 1, Dec. 2000, doi: 10.1006/abio.2000.4836.

[7] C. J. M. Pruitt, K. Benham, B. Bandyopadhyay, and D. J. Goebbert, 'Fragmentation of deprotonated nitrophenol anions', *Chemical Physics Letters*, vol. 614, pp. 192–198, Oct. 2014, doi: 10.1016/j.cplett.2014.09.038.
